# Early Components of the Complement Classical Activation Pathway in Human Systemic Autoimmune Diseases

**DOI:** 10.3389/fimmu.2016.00036

**Published:** 2016-02-15

**Authors:** Katherine E. Lintner, Yee Ling Wu, Yan Yang, Charles H. Spencer, Georges Hauptmann, Lee A. Hebert, John P. Atkinson, C. Yung Yu

**Affiliations:** ^1^Center for Molecular and Human Genetics, Division of Pediatric Rheumatology, Department of Pediatrics, Nationwide Children’s Hospital, The Ohio State University, Columbus, OH, USA; ^2^Laboratoire d’Immuno-Rhumatologie Moleculaire, INSERM UMR_S 1109, LabEx Transplantex, Faculté de Médecine, Université de Strasbourg, Strasbourg, France; ^3^Division of Nephrology, College of Medicine, The Ohio State University, Columbus, OH, USA; ^4^Division of Rheumatology, Department of Medicine, Washington University School of Medicine, St. Louis, MO, USA

**Keywords:** systemic lupus erythematosus, complement C1q, complement C4, autoimmune diseases, complement C2, complement C1s, complement C1r, classical pathway

## Abstract

The complement system consists of effector proteins, regulators, and receptors that participate in host defense against pathogens. Activation of the complement system, via the classical pathway (CP), has long been recognized in immune complex-mediated tissue injury, most notably systemic lupus erythematosus (SLE). Paradoxically, a complete deficiency of an early component of the CP, as evidenced by homozygous genetic deficiencies reported in human, are strongly associated with the risk of developing SLE or a lupus-like disease. Similarly, isotype deficiency attributable to a gene copy-number (GCN) variation and/or the presence of autoantibodies directed against a CP component or a regulatory protein that result in an acquired deficiency are relatively common in SLE patients. Applying accurate assay methodologies with rigorous data validations, low GCNs of total C4, and heterozygous and homozygous deficiencies of C4A have been shown as medium to large effect size risk factors, while high copy numbers of total C4 or C4A as prevalent protective factors, of European and East-Asian SLE. Here, we summarize the current knowledge related to genetic deficiency and insufficiency, and acquired protein deficiencies for C1q, C1r, C1s, C4A/C4B, and C2 in disease pathogenesis and prognosis of SLE, and, briefly, for other systemic autoimmune diseases. As the complement system is increasingly found to be associated with autoimmune diseases and immune-mediated diseases, it has become an attractive therapeutic target. We highlight the recent developments and offer a balanced perspective concerning future investigations and therapeutic applications with a focus on early components of the CP in human systemic autoimmune diseases.

## Activation of the Complement System

The complement system is a humoral recognition and effector system that facilitates in the elimination of invading pathogens. The activation pathways of the complement system converge at C3 and progress to the formation of the membrane attack complexes (MAC or C5b–C9) on a target membrane. The cascades of complement system are mediated and adjusted according to the type of initiator and the microenvironment in which complement activation is occurring. Proteins of the complement system cooperate and coordinate to differentiate among invading microbes, immune complexes, apoptotic cells, cellular debris, and physiologic host cells (1–4).

**Figure 1:**
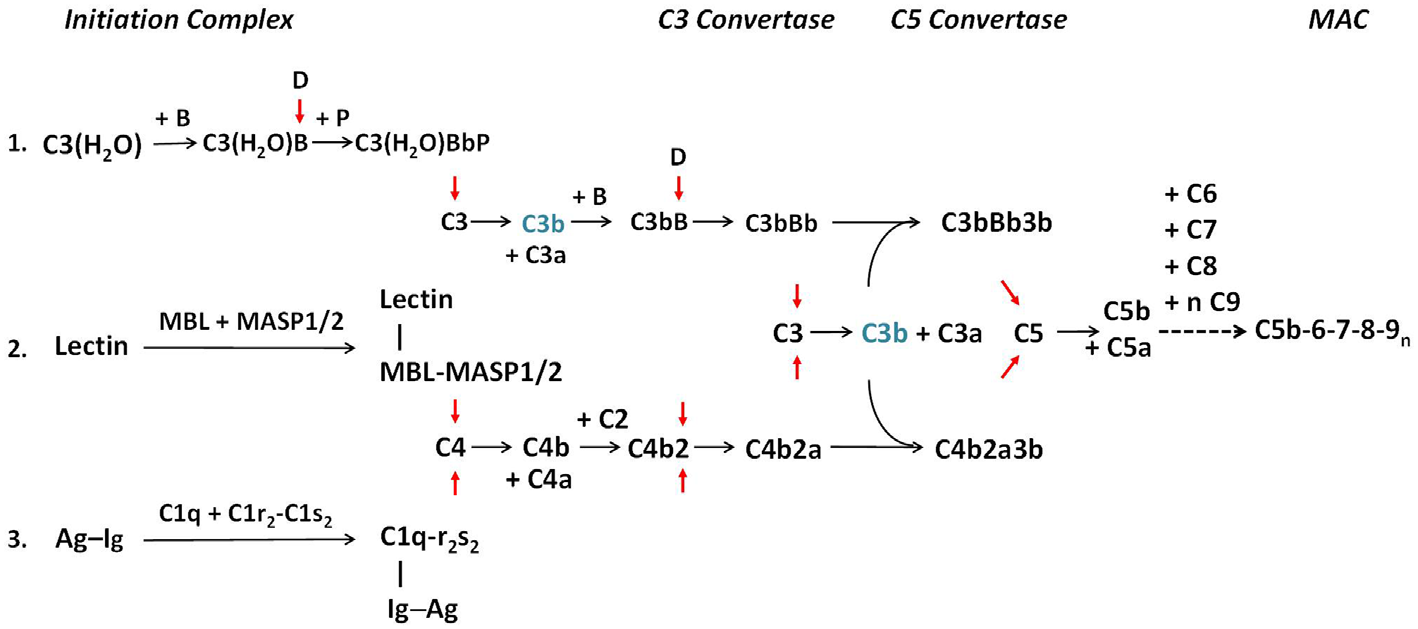
**The activation pathways of the complement system**. The three activation pathways of the complement system are shown according to evolution and physiologic sequences. **Pathway 1** is known as the alternative pathway. It is activated through a tick-over mechanism because of continuous hydrolysis of the thioester bond in C3, which enables the formation with factor B to form a weak C3 convertase. **Pathway 2** is known as the MBL or lectin pathway. It is initiated through the binding of mannan-binding lectin (MBL) or ficolin to arrays of simple sugar molecules in glycosylated antigens on microbes. This is a pattern recognition mechanism characteristic of the innate immune system. **Pathway 3** is known as the classical pathway and is initiated through the binding of specific antibodies IgM or IgG to antigens. It is an effector arm of the humoral adaptive immune system. Each activation pathway engages the formation of a multi-molecular initiation complex, followed by the assembly of a C3 convertase and a C5 convertase for activation of C3 and C5, respectively and culminates in the formation of membrane attack complexes (MAC) on the target membrane. All three pathways can be amplified through a positive feedback mechanism, as C3b (in blue) generated by any C3 convertase can feed to the alternative pathway through association with factor B to form new C3 convertase after activation by factor D (pathway 1). Anaphylatoxins C3a and C5a are produced during the activation process. For brevity, by-products generated during the activation of C2 and factor B are not shown. Red arrows show activation of component proteins through cleavage by serine proteases. A dotted horizontal arrow denotes multiple steps are involved in the formation of the membrane attack complex. Early components of the classical pathway C1q, C1r, C1s, and C4 are engaged in the differentiation of immunity and autoimmunity, as genetic or acquired deficiency in any of these components are linked to pathogenesis of SLE. Complement C2 is also involved in the protection against autoimmunity but its effect size is smaller [modified from Ref. (2)].

Three distinct pathways trigger cascades of activation through proteolysis of zymogens or precursors present in the circulation (Figure 1). The classical pathway (CP) is predominantly triggered by IgM or IgG immune complexes. The formation of antigen–antibody complexes exposes binding sites for C1q on Fc-regions of immunoglobulins, triggering the assembly and activation of the multi-molecular C1 complex, C1q–C1r_2_–C1s_2_ (pathway 3, Figure 1). Conformational changes in the C1 complex are induced upon binding of C1q to antibody, leading to activation of the serine protease subunits C1r and then C1s. As a result, C1s next activates C4 and C2, leading to the formation of the CP C3 convertase (abbreviated C4b2a). Following the early components activation, later components of the complement cascade form the MAC, which perturbs membranes, including the creation of pores across the target membrane, inducing cell lysis, loss of cytoplasm, and osmotic shock.

In the mid-1950s, Pillemer and colleagues of Case Western Reserve University observed that complement activation could occur in the absence of a specific antibody (5). The existence of such an “*alternative”* pathway (AP) of activation was challenged but was confirmed more than two decades later (6). Specific protein factors involved in this AP are named factors, such as factor B, factor D, factor H (FH), and factor P (properdin). This pathway is initiated by a “tick-over” mechanism, in which a small proportion of complement C3 in the circulation is continuously hydrolyzed at slow rate (~1–2%/h) by water to form C3(H_2_O). C3(H_2_O) binds to factor B, which is activated by factor D, to form C3(H_2_O)Bb. C3(H_2_O)Bb accordingly acts as a relatively labile C3 convertase, constantly initiating C3 cleavage. Properdin stabilizes the short-lived C3 convertase. Under the appropriate circumstances, a C5 convertase (C3bBbP) is formed, and the cascade progresses to MAC formation on a foreign cell surface, similar to that of the CP (pathway 1, Figure 1). The binding of P to C3bBb on a microbial (or protected) surface will stabilize and protect the convertase from inactivation by regulatory proteins, thereby enhancing the convertase activity. The AP actually represents an ancient mechanism of innate immune host defense. The tick-over mechanism of complement activation enables a continuous surveillance for the host, executing the first line of defense against foreign invaders. With the development of a circulatory system, a system of host defense that both worked in seconds and was pathogen-destructing became mandatory.

A third pathway of complement activation involves the specific pattern recognition of biomolecules. One strategy for organisms to achieve species-specific diversity is by modification of biomolecules such as glycolipids and glycoproteins with different complexities of sugars. Typically, carbohydrate moieties on glycoproteins among vertebrates consist of complex sugars with secondary modifications (biantennary type) and ending with sialic acids. By contrast, the carbohydrate moieties in prokaryotes generally consist of simpler polymers of saccharides such as mannose. Pattern recognition of biomolecules is a universal theme of innate immunity. This pathway of complement activation is initiated by the binding of pattern recognition molecules including mannan-binding lectin (MBL) or ficolins to a bacterial membrane that express arrays of simple carbohydrates such as mannose and *N*-acetylglucosamine (7, 8). Such binding triggers the assembly of MBL/MASP2 and ficolin/MASP2 or MBL/MASP1 and ficolin/MASP1 complexes (pathway 2, Figure 1). MASP2 and MASP1 are both serine proteases. MASP2, associated with MBL or ficolin, activates both C4 and C2. As a result, a C3 convertase identical to that generated by the CP is formed.

Thus, all three complement activation pathways pass through the focal point on the activation of C3 to C3a and C3b, and then C5a and C5b, leading to the assembly of sublytic or lytic complexes on target membrane. It is noteworthy that all three activation pathways can be amplified by the positive feedback mechanism of the AP. In addition to cell lysis, effects of complement activation include opsonization to enhance phagocytosis of target cells, clearance of apoptotic bodies, solubilization and removal of immune complexes, stimulation of cytokine production, and anaphylatoxin-mediated effects. To summarize, the complement system has been designed in evolution primarily to activate on the membranes of bacteria and certain viruses. Opsonization via C3b and cellular activation via the anaphylatoxins C3a and C5a are its two primary functions.

## Regulation of the Complement System

Because the activated complement components C4b, C3b, and C5b67 can attach to any nearby cell surfaces including host cells, regulatory mechanisms have evolved to contain complement activation to damaged self and foreign targets. Inherently, all activated complement proteins spontaneously undergo intrinsic decay or inactivation when not stabilized by other pathway components or factors. In addition, several regulatory proteins in plasma or on the cell membrane can dissociate (decay) multi-molecular (activated) complexes and also proteolytically degrade the anchor proteins such as C4b and C3b. Upon initiation of classical or lectin pathways, C1-inhibitor (C1-INH) is a serine protease inhibitor that mimics the substrates for C1s in the C1 complex, and MASP2 or MASP1 in the MBL or ficolin complex. C1-INH forms a complex with activated C1r and C1s leading to the dissociation of the enzymatic C1r/C1s subunit from the recognition C1q subunit and preventing further activation of C4 and C2.

Importantly, there are also key strategies of regulation that act upon the assembly and stability of the C3 convertases. Fluid phase proteins, C4b-binding protein (C4bp) and FH and membrane proteins complement receptor 1 (CR1) and decay accelerating factor (DAF) all dissociate the recognition and enzymatic subunits of C3 convertases. Moreover, C4bp, CR1, and membrane cofactor protein (MCP) serve as cofactor proteins for factor I-mediated degradation of C4b, while FH, CR1, and MCP each serves as a cofactor for the factor I-mediated proteolysis of C3b. Notably, C4bp and FH recognize exposed host glycoproteins with glycosaminoglycans and sialic acids. The presence of such regulatory molecules on self-surfaces, but absence from most foreign particle surfaces, allows the regulators to prevent activation on host tissues while restricting complement activity to designated, foreign targets. Dysfunctional or uncontrolled complement activation can lead to destruction of body cells, overt release of inflammatory mediators, and tissue damage, as evidenced by clinical complications experienced by systemic lupus erythematosus (SLE) patients.

## Complement in Systemic Lupus Erythematosus

### Serum C4 and C3 Levels

**Figure 2:**
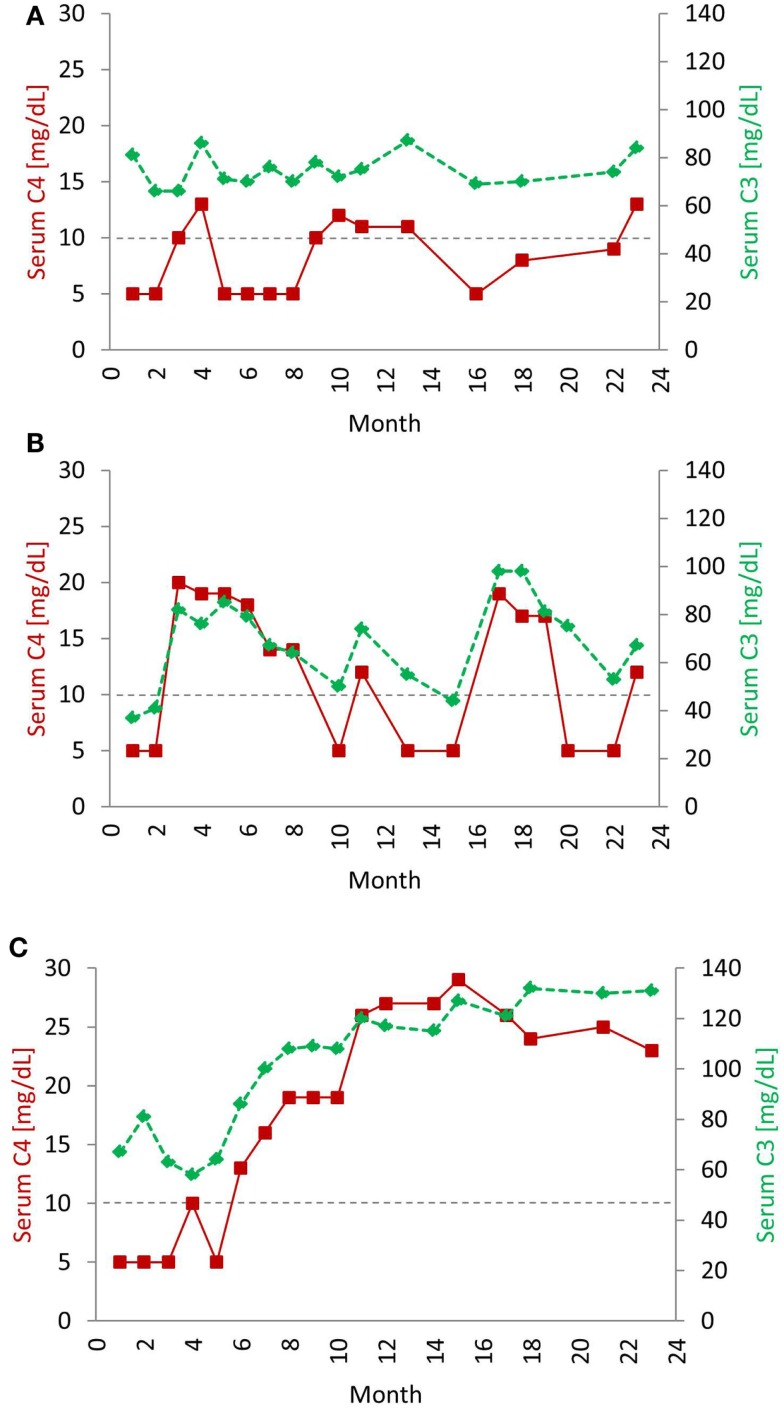
**Typical serial serum protein profiles of complement C4 and C3 in human SLE patients**. Serum C4 (red, solid line) and C3 (green, dashed line) protein levels tend to go up and down together in most SLE patients. The horizontal dotted line indicates the low level of serum C4 (<10 mg/dL), below which usually requires clinical attention. The profiles shown are taken from three individual patients over a 24-month period and represent three common profiles typically observed in SLE patients. In the first profile **(A)**, levels of C4 and C3 were chronically low. In some patients, even if C3 levels rose to normal range, C4 levels remained low. Patients with this profile are often characterized by low copy-number of C4 genes. **(B)** The second profile had frequent and parallel fluctuations of serum C3 and C4. Patients with this profile had active disease, and low C3 and low C4 roughly correlated with disease activity. In the third profile **(C)**, C4 and C3 protein levels stayed in the normal range most of the time, except at the time of diagnosis and during a disease relapse. Patients with this profile had relatively inactive disease. Patients with the second and third profiles have normal gene copy-number of total C4 but may have a heterozygous deficiency of C4A [modified from Reference (12)].

Systemic lupus erythematosus patients commonly present with evidence of complement consumption leading to low serum levels of C4 and C3 (9, 10). Initially, up to one-half of SLE patients will have low C4 and C3. In most established patients, serum C4 levels are biomarkers for lupus disease activity; low levels correlate with a flare, while normal levels correspond with remission (11). Longitudinal studies of serum C4 protein levels in SLE patients revealed different expression profiles characterized by three distinct groups (Figure 2) (12). The first group exhibited persistently low C4 levels throughout the course of the study, and many of these patients had a low copy number of C4 genes. The second group featured periodic fluctuations of C4 that paralleled disease activity while the third group had normal C4 levels most of the time. The typical pattern in active SLE patients is that both C4 and C3 are low simultaneously. However, exceptions occur. C3 levels are usually three- to sixfold higher than C4 levels; therefore, consumption of complement by immune complexes could reduce C4 below normal but leave C3 in the normal range. With a positive response to treatment, both C4 and C3 levels will rise. As noted, up to one-half of SLE patients will present with serum C4 and C3 in a normal range, which obviously does not rule out a lupus diagnosis. The CH50 and AP50 measure the lysis of red blood cells by the respective pathway and thus are functional tests. Furthermore, *in vivo* complement activation can also be assayed by testing for complexes or split products formed during activation (3).

Copy-number variation (CNV) of C4 can affect serum C4 protein concentrations. In an American Caucasian populations, about 60% of individuals have four copies of the C4 gene, 28.5% have three (or less), and 12.5% have five (or more). In lupus, the number of patients with three or less C4 genes may increase to 42.2% (13). If an individual has low copy of C4 genes, the baseline C4 antigenic level may be 12–18 (~6 to 8 mg/dL per copy of a C4 gene). In this situation, it does not take much activation to lower the C4 out of the normal range. Additionally, a subject’s body mass index (BMI) is positively associated with serum C4 or C3 protein concentrations (14, 15). All things considered, the care of each patient must be individualized. Repeated, longitudinal serum measurements of C3 and C4 are usually clinically utilized.

### Cell-Bound C4d as a Biomarker of Complement Activation for Humoral Immunity, Alloreactivity, and Autoimmunity

In the past decade, cell-bound levels of processed complement activation products (CBCAPS), especially erythrocyte-bound C4 (E-C4d), has been proposed to assist in the diagnosis and clinical monitoring of SLE (16, 17). E-C4d is a stable, proteolytic end fragment of C4, which is covalently bound to the surface of erythrocytes following activation of the CP or MBL pathway. On cell surfaces, the activated C4b is processed to the cell-bound C4d with the release of soluble C4c through factor-I mediated proteolysis in the presence of a cofactor (i.e., CR1 or MCP on the plasma membrane or C4bp from plasma). In 2004, Manzi et al. found that erythrocytes from SLE patients had markedly higher levels of E-C4d when compared to healthy controls or patients with other diseases (16). Additional studies explored the utility of CBCAPS on T and B lymphocytes, platelets, and reticulocytes in SLE (18–21). The levels of C4d bound to the membrane of these cells were significantly higher in SLE than healthy controls or patients with other diseases. In a study of 304 SLE patients, 285 patients with other rheumatic diseases and 205 healthy controls, Putterman and colleagues reported that CBCAPS on erythrocytes or B cells had higher sensitivity than standard complement levels (serum C3 and C4) and anti-dsDNA measurements when distinguishing between SLE and non-SLE, suggesting that CBCAPS could be more specific and sensitive biomarkers for diagnosis and prognosis of SLE (17). However, given the relative simplicity and low cost of serum C4 and C3 measurements, it remains to be seen how assessment of cell-bound C4d and C3d will contribute to the clinical care of patients with SLE.

Historically, cell-bound complement activation proteins, particularly those of C4d, have offered clues to several important discoveries. Between 1960 and 1990, it was found that blood group antigens, Chido (Ch) and Rodgers (Rg), were polymorphic variants of complement C4 (22–24). Alloantibodies generated against Ch/Rg antigens after blood transfusion in certain recipients were *mostly* directed against polymorphic amino acids present in the C4d region of C4B and C4A proteins, respectively. The mapping of the Ch/Rg variants to the HLA contributed to the understanding of MHC genetics. The polymorphisms of C4A and C4B protein allotypes are readily demonstrated by immunofixation of EDTA-plasma resolved by high voltage, agarose gel electrophoresis based on gross differences of electric charge of protein molecules resulting in variations in electrophoretic mobilities (25–27). Using an assay that involved an overlay of “sensitized” sheep red blood cells on the described C4-allotyping gels, it was found that C4B proteins are functionally 4–10 times more active on the hemolysis of sheep red blood cells (26, 28, 29). While most C4A are associated with Rg antigens and C4B with Ch antigens, reverse associations such as C4A1 with Ch and C4B5 with Rg were demonstrated (23, 30). It was the cloning and sequencing of those functional and serological variants that enabled the identification of specific isotype residues at positions 1120–1125, PCPVLD for C4A and LSPVIH for C4B, encoded by exon 26 of the C4 gene (31, 32). The major epitopes for Rg and Ch blood groups were mapped to positions 1207–1210, VDLL for Rg and ADLR for Ch, encoded by exon 28 of C4 genes (32). Detailed characterization of C4A and C4B genetic polymorphisms unraveled a surprising phenomenon: CNVs and gene size dichotomy among human subjects with specific distribution patterns among different racial populations (13–15, 33, 34).

In parallel to the discovery of common CNV for C4A and C4B genes that contribute to quantitative and qualitative phenotypic diversities of innate immunity and associations with autoimmune diseases, another far-reaching observation related to application to cell-bound C4d occurred in the field of organ transplantation. The deposition of C4d on the endothelium of capillaries for renal, heart, and lung grafts is recognized to be a diagnostic biomarker or “immunohistochemical imprint” of alloantibody-mediated complement activation that leads to graft rejection. In transplant recipients, preexisting or *de novo* donor specific *allo*antibodies binding to graft cells may activate complement and cause graft injury. In acute and chronic rejections of renal grafts, peritubular capillary (PTC) deposition of C4d occurred in about 30% of biopsy specimens and detection of diffuse PTC-C4d often associates with poor renal graft outcome. Reviews of C4d-deposition on graft capillaries as a result of humoral alloreactivity can be found in Ref. (35–37).

## Hereditary Genetic Deficiencies of Early Complement Components in SLE

**Figure 3:**
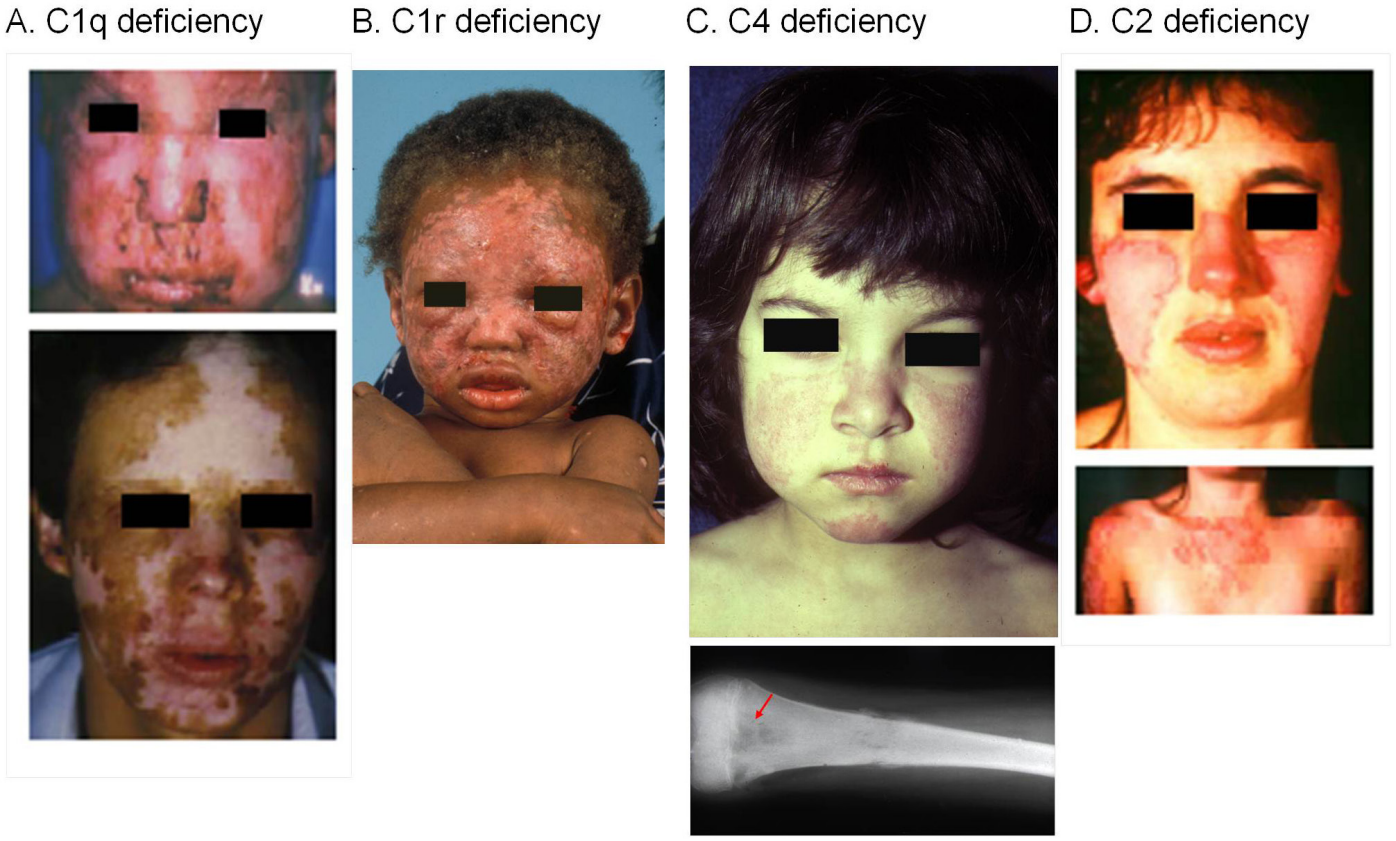
**SLE patients with a homozygous deficiency of early components for the classical pathway of complement activation**. Severe cutaneous lesions are common clinical presentations in SLE patients with a complete complement deficiency. **(A)** A homozygous C1q-deficient male child with cutaneous infection (upper panel) and with discoid lupus erythematosus and scarring lesions on face when he was 22 years old (lower panel). **(B)** A male child with discoid lupus at 16-month old with homozygous C1r-deficiency. This patient experienced generalized seizures, developed a scissoring gait with toe walking, spasticity and weakness of the legs. At 18 years old, he was diagnosed with class IV lupus nephritis and progressed to end-stage renal disease. **(C)** A complete C4-deficient girl at 3 years old with butterfly rash and cheilitis (upper panel), and osteomyelitis of the femur at 10 years old (lower panel). This patient died at age 12 because of pulmonary infection and cardiovascular failure. **(D)** A homozygous C2-deficient young woman with acute cutaneous lupus erythematosus. The upper panel shows the butterfly rash, and the lower panel shows photosensitive lesions on sun-exposed areas [adopted from Ref. (40–43)].

Deficiencies or genetic polymorphisms of early complement components are strongly associated with increased risk of developing SLE or a lupus-like disease (Figure 3). Complement deficiencies are hypothesized to be associated with increased susceptibility of SLE for several reasons. Functionally, the role of complement includes the identification, opsonization, and proper disposal of apoptotic cells and immune complexes formed regularly between antibodies and foreign or self-antigens (1, 38). An inability to efficiently clear apoptotic cells could render them a source of autoantigens and thereby drive autoantibody production. Impaired clearance of immune complexes and “self” debris provides a logical explanation for complement deficiency in the induction of SLE (39). While there are multiple complement pathways to assist the host with clearance of these types of materials that accumulate continuously in healthy subjects, the CP is essential through at least C4, and to a lesser degree C2, to properly handle and dispose of immune complexes and apoptotic debris.

Another hypothesis that attempts to explain the association of complement deficiency with SLE suggests that the complement system is involved in immune tolerance (44). In other words, early components of the CP are engaged in the “cross-talk” to the adaptive immune system to achieve tolerance against self-antigens, or in the discrimination of “self” versus “non-self.” A complement deficiency that results in a breach of self-tolerance provides a reasonable explanation for association with SLE (45, 46). Normally, complement receptor 1 (CR1/CD35) and complement receptor 2 (CR2/CD21) on follicular dendritic cells of peripheral lymphoid tissues (such as the spleen and lymph nodes) bind to and deliver self-antigens coated with complement fragments to the autoreactive B-cells, which are anergized or kept away from germinal center reactions. Previously, it was demonstrated in mouse models that complement was necessary for the elimination of self-reactive lymphocytes during the maturation of the immune system (46). Specifically, Prodeus et al. showed that deficiency of either CD21/CD35 or of C4 in a well-defined mouse model of peripheral tolerance resulted in high titers of anti-nuclear antibodies (ANAs) and a severe lupus-like disease.

These two concepts of debris clearance and regulation of self-tolerance are not exclusive and likely overlap, or together are responsible for the development of SLE in humans deficient in early components of the CP.

In the following sections, we will describe genetic complement deficiency states associated with SLE for each component of the early CP (C1q, C1r, C1s, C4, and C2).

### Complement C1q Deficiency

Three different genes (C1qA, C1qB, C1qC) closely linked on the short arm of chromosome 1 encode for the C1q protein, which is composed of 18 polypeptide chains. Inter-chain disulfide bonds are formed via the cysteine residue in the N-terminal region of each chain. Following the N-terminal region is a collagen-like region (CLR) of ~81 residues. One A chain and one B chain form a heterodimer during biosynthesis, and two C chains form a homodimer, both through disulfide linkages via conserved cysteine residues. Two A–B heterodimers associate with one C–C homodimer to form a hexameric structure, of the composition ABC–CBA. Three of these hexamers, with a total of 18 polypeptide chains together, form the tulip-like structure of C1q with a collagenous tail and six globular regions, each with globular heads ghA, ghB, and ghC.

The CP of activation is initiated by the C1 complex, of which C1q is the first subcomponent. When C1q in the C1 complex binds to IgM or IgG present in an immune complex, a binding site of C1r/C1s is exposed, allowing further activation of the complement pathway (47–49). C1q is an important opsonin to promote phagocytosis of apoptotic cells or debris, which can be archived directly without complement activation through binding at the collagenous region of C1q to calreticulin (CRT) in apoptotic cell blebs and to CD91 on phagocytes; or *indirectly* with activation of the CP as C1q binds to CRP ligated to phosphorylcholine/phosphatidylserine or to SAP ligated to fragmented chromatin from apoptotic cells, generating processed products C4b, C3b, iC3b, C3dg, and C3d that are ligands for CR3 and CR4 on myeloid cells to initiate phagocytosis.

The number of reported cases of homozygous deficiency of C1q has increased to 74 (3, 50–52). Among the reported C1q-deficient subjects, the median age of (any) disease onset was 6 years. The clinical presentations among C1q-deficient patients varied considerably, but the two common observations were: (a) SLE or lupus-like disease in 88% and (b) recurrent bacterial infections in 41% (40, 52). Some patients (17%) died at a young age secondary to septicemia. Among the C1q-deficient patients with SLE or lupus-like disease, cutaneous disorders, especially photosensitivity, were prominent with a frequency of 84%. Glomerulonephritis and neurologic disease affected about 30 and 19% of patients, respectively. Oral ulceration occurred in 22% and arthritis/arthralgia in 16%. Immunologically, most C1q-deficient patients had normal serum levels of complement C4 and C3, high frequency of ANAs (particularly anti-Ro/SSA) but a low frequency of anti-dsDNA.

Several different causative mutations have been identified in patients with complete C1q deficiency. A variety of mutations (including non-sense, frameshift indels, and splice site) result in the absence of biosynthesis of one of the three C1q (A, B, or C) chains. Other mechanisms have been identified by which C1q protein is defective in secretion, structure, or function. In Table 1, the molecular basis of the genetic mutations leading to complete absence or complete functional deficiency of C1q protein for which genetic information is reported, as well as the accompanying clinical manifestations.

**Table 1:** **Complete deficiency of A, B, or C chain genes of C1q**.

	Location	Mutation	Molecular defect	Age of onset (years), sex, ethnicity	Clinical presentations	Reference
1	C1qA	M1R	Start codon mutation; no detectable protein	nk, M, African-American^a^	SLE	(53)
2	C1qA	M1R	As above	nk, M, African-American^a^	Lupus, premature death	(53)
3	C1qA	Q208X	Nonsense mutation	10, M, Turkish	History of ear and oral infections, recurrent skin lesions, premature death at age 10 from septicemia	(54–56)
4	C1qA	Q208X	As above	4, F, Turkish^b^	Malar rash, stomatitis, ANA, premature death at age 6 from sepsis	(56, 57)
5	C1qA	Q208X	As above	6, F, Turkish^b^	Facial swelling, hematuria	(56, 57)
6	C1qA	Q208X	As above	nk, F, Turkish	Asymptomatic at age 22	(58)
7	C1qA	Q208X	As above	3, F, Turkish^c^	SLE, glomerulonephritis, arthralgias, photosensitivity, anti-Ro autoantibodies	(59)
8	C1qA	Q208X	As above	15, F, Turkish^c^	Photosensitive rash, microscopic hematuria, IgA nephropathy	(59)
9	C1qA	Q208X	As above	4, M, Turkish	SLE-like disease, meningitis, pneumonia, meningococcal sepsis, ANA	(60)
10	C1qA	Q208X	As above	1, M, Turkish	Rash, recurrent upper respiratory tract infections, low ANA	(61)
11	C1qA	Q208X	As above	1, M, Iraqi	Erythematous rashes, otitis media, glomerulonephritis, fatigue, photosensitivity, ANA	(52)
12	C1qA	W216X	Nonsense mutation	0.5, F, Sudanese^d^	SLE, cutaneous lupus, bacterial meningitis, ANA	(52)
13	C1qA	W216X	As above	3, M, Sudanese^d^	SLE, cutaneous lupus, bacterial meningitis, bacterial keratitis, polyarthritis, ANA	(52)
14	C1qA	1-bp deletion; Q64X	Frameshift mutation → premature stop codon	3, M, Caucasian	Photosensitivity, malar rash	(52)
15	C1qB	Point mutation (RFLP analysis only)	Premature stop codon; functionally deficient protein	4, M, Pakistani	SLE-like disease, history of fever, glomerulonephritis, discoid facial lesions, ANA, premature death at age 8	(62, 63)
16	C1qB	G42D	Glycine mutation; LMW C1q; complete functional deficiency	16, F, Moroccan^e^	SLE, arthralgia	(64, 65)
17	C1qB	G42D	As above	23, M, Moroccan^e^	SCLE, ANA, anti-Sm autoantibodies	(64, 65)
18	C1qB	G42D	As above	3, M, Moroccan^e^	SLE, ANA, anti-ds DNA autoantibodies, thrombocytopenia, growth retardation	(64, 65)
19	C1qB	G42D	As above	nk, M, Moroccan^e^	Asymptomatic at age 42	(64, 65)
20	C1qB	G244R	Glycine mutation; no detectable protein	3, F, Inuit^f^	DLE, photosensitive malar rash, ANA, recurrent skin and mucosal lesions	(66)
21	C1qB	G244R	As above	14, F, Inuit^f^	SLE, ANA, arthritis	(66)
22	C1qB	G244R	As above	2, F, Inuit^f^	Lupus erythematosus, vasculitis, pneumonia, ANA	(66)
23	C1qB	G63S	Missense mutation; C1q unable to associate with C1r and C1s	20, M, Arabian	SLE, CNS involvement, recurrent infections; premature death due to bacteria-induced septic shock	(67)
24	C1qB	6251A > C	Splice site mutation; no detectable protein	2, M, Caucasian	Recurrent upper airway infections, history of fever and seizures	(68)
25	C1qB	187G > T	Splice site mutation; complete functional deficiency	4, F, Japanese	DLE, history of fever, facial erythema, joint pain, oral ulcerations	(69)
26	C1qC	G34R	Glycine mutation; LMW C1q; complete functional deficiency	4, F, Indian^g^	DLE, photosensitivity, ANA	(70)
27	C1qC	G34R	As above	0.8, M, Indian^g^	Umbilical sepsis, erythematous rash, ANA, parotitis, anti-Ro autoantibodies	(70)
28	C1qC	G34R	As above	0.5, F, Arabian	SLE-like disease with CNS involvement, recurrent bacterial infections, ANA, anti-dsDNA autoantibodies, hyper-IgM syndrome	(71)
29	C1qC	G34R	As above	21, F, Caucasian	Adult-onset of SLE-like disease, history of fever, oral ulcerations, bacterial meningitis, ANA, anti-Ro autoantibodies	(72)
30	C1qC	R69X	Nonsense mutation	9, F, Caucasian	Severe SLE, with cutaneous and CNS involvement, ANA, anti-Sm and anti-Ro autoantibodies, cytomegalovirus retinitis, premature death at age 28 from CNS involvement	(70, 73)
31	C1qC	R69X	As above	10, M, Kosova	Malar and discoid rash, ANA, oral ulcerations	(52)
32	C1qC	G76R	Glycine mutation; no detectable protein	8, F, Turkish	Recurrent meningitis, pneumonia	(74)
33	C1qC	delC43 fs108X	Frameshift premature stop codon at 108	1, M, Yugoslavian^h^	SLE-like disease, photosensitivity, butterfly rash, glomerulonephritis, ear infections, ANA, anti-Sm and anti-Ro autoantibodies	(70, 75)
34	C1qC	delC43 fs108X	As above	3, M, Yugoslavian^h^	SLE-like disease, cutaneous vasculitis, ANA	(70, 75)
35	C1qC	1bp deletion → 83X	Frameshift mutation premature stop codon at 83	6, F, Pakistani	Erythematosus rash, recurrent urinary and respiratory infections, ANA, anti-Sm autoantibodies	(76, 77)

*^a–h^Individuals marked with matching superscripts are from the same family*.

*nk, age of onset is not known; DLE, discoid lupus erythematosus; SCLE, subacute cutaneous lupus erythematosus; SLE, systemic lupus erythematosus; LMW, low molecular weights; ANA, antinuclear antibodies*.

Most of the causative mutations associated with homozygous C1q genetic deficiency are the result of a consanguineous marriage. The affected patients are likely close descendants of ancestors carrying the specific deleterious mutation. Surprisingly, screening of large SLE cohorts from those countries with reported cases of C1q deficiency to determine the prevalence of C1q deleterious mutations have yielded negative results (59, 78), suggesting that the mutations are “private” and rare but with very large effect size, as documented in many complex diseases (79, 80).

### Complement C1r and C1s Deficiency

The genes for human C1s and C1r are located on the short arm of chromosome 12 (81). According to bioinformatics studies and previous publications (82), C1r and C1s are configured in a tail-to-tail orientation with their 3′ ends separated by ~9 kb. The DNA sequence for the genomic region harboring human C1s and C1r coding sequences is still incomplete in the Reference Genome (Annotation release 106, January 2015) and consists of gaps.

C1r and C1s are paralogous proteins that share 38% identity and 55% similarity. Each mature protein is a proenzyme consisting of six distinct modules: two CUB domains separated by an EGF domain with a binding site for Ca^2+^, followed by two complement controlling protein repeats CCP1 and CCP2, a linker segment and then a chymotrypsin-like serine protease domain SP at the carboxyl-terminus region. In circulation, C1r and C1s are proenzymes that exist as a tetrameric structure, C1s–C1r–C1r–C1s, which assembles in the presence of Ca^2+^ with C1q to form the multi-molecular C1 complex. Upon activation of C1q (e.g., through binding of its globular heads to the Fc-regions of IgG or IgM in an immune complex), the tetramer interacts with the hinge region of C1q to form the activated C1 complex. Autoactivation of the two C1r by proteolytic cleavages between Arg-463 and Ile-464 is followed by activation of C1s by proteolysis between Arg438 and Ile-439, which release the enzymatic activity of the nascent C1 complex. The C1s in this C1 complex activates C4 and then C2 which together form the CP C3 convertase, C4b2a.

Deficiencies in subcomponents C1r and C1s were among the earliest reports linking complement deficiency with human glomerulonephritis or a lupus-like disease (83–85). A total of 20 cases of C1r and/or C1s deficiencies have been reported, which include 12 cases of C1r deficiency from eight families and eight cases of C1s deficiency from five families. Among the C1r-deficient patients, there was a consistent reduction in the serum protein levels of C1s to 30% of its normal level, but highly *elevated* serum protein levels of C4, C2, and C1-inhibitor (200–400% of their corresponding normal ranges). C3 was also elevated by ~50%, but C1q levels were normal. A similar phenomenon was observable among C1s-deficient patients; C1s-deficient patients had greatly reduced serum levels of C1r, markedly elevated levels of C4, C2, C1-inhibitor, and C3, and normal levels of C1q.

Among the C1r/C1s deficient subjects, all but three had recurrent bacterial, viral, or fungal infections (85%), and many patients died at young age because of a severe infection. Thirteen subjects (65%) developed SLE or a lupus-like disease. The prevalence of ANA among these patients was about 60%. Mortality at young age from infections likely explains the slightly lower frequency of lupus disease association compared to C1q deficiency. Most C1r/C1s deficient patients had severe cutaneous lesions (Figure 3). Eight patients (40%) had renal disease due to lupus nephritis. Such presentations underscore the inter-dependence of C1r and C1s in sustaining a stable tetrameric structure that would otherwise be susceptible to a high turnover rate. A deficiency of C1r or C1s prevents the formation of the C1 complex and diminishes the need for engagement of C1-inhibitor and other regulators of complement activation. When C1 is not functional, the CP is not activated, and consumption of C4, C2, and C3 is greatly reduced, resulting in *high* levels of these proteins in the circulation (41). This and other results strongly indicate a chronic turnover of component proteins for the CP (86).

The molecular defects leading to C1r or C1s deficiency have been determined in one case of C1r deficiency and seven cases of C1s deficiency (Table 2). Relative to C1r deficiency, the defect was a homozygous C to T substitution in exon 10 resulting in the R380X non-sense mutation in the second CCP domain, resulting in no detectable protein in the serum (41). The proband developed SLE at 3 months of age and presented with reduced levels of C1s (similar to other C1r-deficient patients), but highly elevated protein levels of C4, C2, and C1 inhibitor.

**Table 2:** **Molecular defects and clinical presentation of complete deficiency for C1s or C1r**.

	Location	Mutation	Molecular defect	Age of onset (years), sex, ethnicity	Clinical Presentations	Reference
1	C1s, Exon 6	Y204X	Nonsense mutation; no detectable protein	7, F, Brazilian^a^	SLE, recurrent infections (pneumonia, septic arthritis, sinusitis), ANA, anti-Sm autoantibodies, arthritis, proteinuria, deposition of IgG and C1q on the glomeruli	(87)
2	C1s, Exon 6	Y204X	Nonsense mutation; no detectable protein	13, M, Brazilian^a^	SLE, arthritis, ANA, anti-Sm autoantibodies, photosensitivity	(87)
3	C1s, Exon 6	Y204X	Nonsense mutation; no detectable protein	nk, M, Brazilian^a^	Asymptomatic at age 20	(87)
4	C1s, Exon 6	Y204X	Nonsense mutation; no detectable protein	nk, M, Brazilian^a^	Asymptomatic at age 10	(87)
5	C1s, Exon 10, Exon 12	4-bp deletion + E597X	Frameshift mutation leading to non-sense mutation; no detectable protein	4, M, Japanese^b^	Virus-associated hemophagocytic syndrome; history of fever; seizures with loss of consciousness leading to premature death at age 7	(88, 89)
6	C1s, Exon 12	G630Q + E597X	Missense mutation; non-sense mutation; truncated protein (functionally inactive) detectable at extremely low levels in serum	13, F, Japanese^b^	History of fever and pain, ANA, seizures, and periods of unconsciousness	(90)
7	C1s, Exon 12	R534X	Nonsense mutation; no detectable protein	2, F, Caucasian	Recurrent malar rash, mild fever, pain and swelling in joints, lupus-like syndrome, Hashimoto’s thyroiditis, autoimmune hepatitis	(91)
8	C1r	R380X	Nonsense mutation; no detectable protein	0.3, M, African American	SLE, discoid lupus rash, diffuse proliferative glomerulonephritis, transverse myelitis	(41)

*^a–b^Individuals marked with matching superscripts are from the same family*.

*nk, age of onset is not known; SLE, systemic lupus erythematosus*.

For C1s-deficiency, several deleterious mutations have been identified. A C→G mutation in exon 6 (Y204X) resulted in a premature stop codon and abrogated any protein production (87). This particular non-sense mutation was homozygous in four siblings, and all showed no detectable C1s protein, but only two developed SLE at the ages of 7 and 13 years. The other two siblings, at ages 10 and 20, did not have clinical symptoms of SLE. Significantly reduced levels of C1r and elevated serum C4 were detected in all four siblings. In another case report, a 4-bp deletion (TTTG) in exon 10 that led to a frameshift and a non-sense mutation in exon 12 (E597X) was detected in a single patient (88, 89). This patient developed unique symptoms including virus-associated hemophagocytic syndrome and died after a long period of a comatose state. A mutation documented in another patient from the same family was a heterozygous G → T mutation in exon 12 leading to E597X and on the other allele, a novel missense mutation G630Q (90). This patient displayed symptoms that were similar to the other, related patient, including fever of unknown origin and short-term disturbances of consciousness. A second C1s genetic variant is a non-sense C → T mutation in exon 12 (R534X) (91). This patient had undetectable serum C1s, normal C1r and C1q, and absence of CH50 activity. The patient was 2 years of age and presented with several autoimmune diseases, including a lupus-like syndrome, Hashimoto’s thyroiditis, and autoimmune hepatitis. Each of these clinical observations and the similarities in autoimmune presentation with C1r/C1s deficiencies highlight the role of a dysfunction in the CP of complement leading to systemic autoimmune disease.

### Complement C4 Gene Copy-Number Variations and C4A or C4B Isotype Deficiency

**Figure 4:**
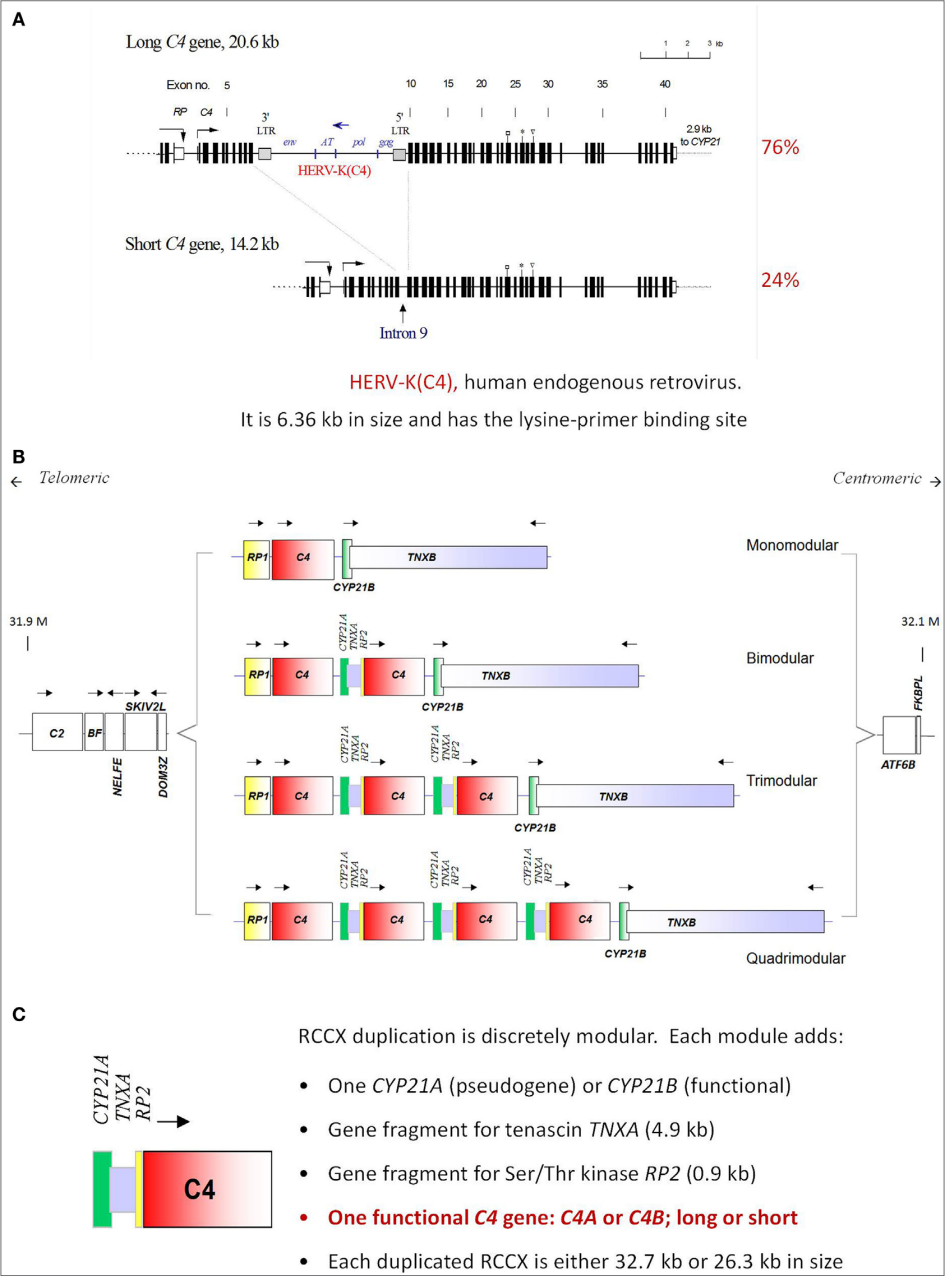
**Gene size dichotomy and gene copy-number variation of complement C4**. A human C4 gene consists of 41 exons coding for a precursor protein of 1744 amino acids including a signal peptide of 19 amino acids. **(A)** There are two forms of C4 genes. The long gene is 20.6 kb, and the short gene is 14.2 kb. In a long C4 gene, an endogenous retrovirus HERV-K (C4), which is 6.4 kb in size, integrated into its ninth intron. Among healthy subjects of European ancestry, 76% of C4 genes belong to the long form and 24% belong to the short form. **(B)** Among European subjects, one to four copies of C4 genes are present in the central region of the major histocompatibility complex (MHC) located on chromosome 6p21.3. Thus, there is a continuous variation in copy number of C4 genes from two to eight copies among different human subjects. **(C)** The duplication of a C4 gene occurs in a modular fashion, with a 0.9 kb fragment of RP (STK19) upstream of complement C4, a full steroid cytochrome P450 21-hydroxylase (CYP21) and a 4.0 kb fragment of the tenascin (TNX) at the downstream region of C4 (known as a RCCX module). The duplication of CYP21 gene can be a pseudogene (CYP21A or CYP21A1P) or an intact functional gene (CYP21B or CYP21A2). Each C4 gene in the RCCX module may either code for an acidic C4A or a basic C4B. Each C4 gene may be either long or short [adopted from Ref. (97–99)].

The complement C4 gene is located in the HLA class III region on the short arm of chromosome 6, telomeric of the C2 gene (92). Remarkably, there are extensive inter-individual gene CNVs for complement C4. Two to eight copies of C4 genes can be present in a diploid genome (93, 94) (Figure 4). Such common CNVs are an uncommon phenomenon in mammalian genetics, as they deviate from the conventional “one-to-one” concept for a gene and a polypeptide/gene product. Here, one to four copies of nearly identical genes co-exist on a single chromosome, thereby creating a gene dosage effect for a quantitative phenotype. Segmental duplications for C4 always include the RP (STK19) gene upstream of C4, and the downstream genes CYP21 and TNX (94). Each C4 gene either encodes for an acidic C4A or a basic C4B protein, with only four amino acid changes (**PC**PV**LD** 1120–1125 for the C4A isotype and **LS**PV**IH** for the C4B isotype), but this results in substantial differences in chemical reactivity for peptide and carbohydrate antigens (28, 29, 31, 95). C4A favors binding to amino groups (i.e., immune complexes) while C4B favors binding to hydroxyl or carbohydrate-rich groups. Approximately 40 protein variants for complement C4 have been documented (25). Technically, it is noteworthy to mention that the differential binding to hydroxyl- or amide-group containing substrates/immune complexes, or hemolytic activities between activated C4A and C4B can be readily demonstrated using purified component proteins or proteins resolved by gel electrophoresis, but not from sera when many regulatory proteins are present (15, 96).

The primary site for C4 biosynthesis is in the liver. However, multiple tissues also synthesize C4, presumably for local consumption, particularly after stimulation by interferon-gamma (100). A thioester bond is present but hidden in native C4. A proteolytic cleavage by activated C1s removes a 74 amino acid C4a peptide and leads to a remarkable change of conformation in C4 (101). Consequently, the protected thioester bond becomes exposed to the exterior. In activated C4B, one of the four isotypic residues (Histidine-1125) serves as a catalyst and facilitates a rapid nucleophilic attack, resulting in formation of a covalent ester linkage between C4B and the target surface (102, 103). In activated C4A, Histidine-1125 is not present, and such a catalytic reaction does not occur. Instead, C4A reacts effectively with an amino group on an immune complex or a protein molecule to form a covalent amide bond. Such a difference in chemical reactivity appears to diversify the functional roles of C4A and C4B in the clearance of immune complexes and the propagation of activation pathways, respectively.

A complete or homozygous genetic deficiency of both complement C4A and C4B has been reported in 28 individuals (42, 104–107). The subjects came from 19 families with different racial backgrounds were characterized by 16 different HLA haplotypes. The female to male ratio was 1:1. SLE or lupus-like disease was diagnosed in 22 (78.6%) of the C4-deficient subjects, and four others had renal disease including glomerulonephritis. Early disease onset, severe photosensitive skin rash, the presence of autoantibodies against ribonuclear protein Ro/SSA, and high titers of ANA were common clinical features of the subjects. Many of the C4-deficient patients also had severe proliferative glomerulonephritis. We have summarized the molecular basis of complete C4 deficiency determined in 15 cases (Table 3).

**Table 3:** **Molecular defects and clinical presentations of complete deficiency of complement C4A and C4B**.

	Location	Mutation	Age of onset (years), sex, race/ethnicity	Clinical presentations	RCCX	HLA	Reference
1	Exon 20	Homozygous 1-bp C-deletion, codon 830; premature stop	2, F, Swedish	SLE-like disease, atypical rash, ANA, rheumatoid factor, persistent exanthem, glomerulonephritis	L (C4A)	A30 B18 DR3	(107–109)
2	Exon 29	*Heterozygous*; identical 2-bp insertion to codon 1232 of all three C4 genes	30, F, Finnish^a^	Malar rash, photosensitivity, polyarthritis, leukopenia, ANA (1/320), anti-Sm (1/1280), weakly positive rheumatoid factor	*LS (C4A-C4B)/L (C4A)*	A2 B39 Cw7 DRB1*1501/A2 B40 Cw3 DRB1*1501	(105)
3	Exon 29	As above	nk, M, Finnish^a^	Photosensitivity	As above	As above	(105)
4	Exon 29; Exon 13	2-bp insertion in codon 1232 of C4A; 1-bp deletion in codon 541 of C4B	9, M, US-French descent^b^	SLE, arthralgia, malar rash; photosensitivity; ANA (1/10240), positive for anti-Sm, anti-U1 ribonuclear protein, anti-cardiolipins; class III nephritis, neurological disease, brain vasculitis; Sjogren’s syndrome, recurrent infections, Raynaud’s phenomenon; died at age 23	LS (C4A-C4B)	A2 B12 DR6	(106)
5	Exon 29; Exon 13	As above	42, M, US-French descent^b^	Discoid rash, polyarthralgias, oral ulcers	As above	As above	(106)
6	Exon 13	GT-deletion in codon 516	10, M, Austrian/Italian	History of fever, macrohematuria, mesangial GN; infection, nephrotic syndrome; membranous GN	L (C4A)	A24 Cw7 B38 DR13	(104)
7	Exon 13	As above	5, M, Austrian/Italian^c^	Renal failure, mesangial GN; skin disease with facial rash; a brother died at 3 with cerebral vasculitis and sepsis	L (C4A)	A24 Cw7 B38 DR13	(104)
8	Exon 13	As above	2, F, Austrian/Italian^c^	SLE, history of fever, skin rash and lesions, oral ulcers, microscopic hematuria, mesangial GN; skin transplant	As above	As above	(104)
9	Intron 28, splice donor	g8127a (GT → AT) both C4B genes	17, M, Austrian/Italian	Henoch-Schoenlein purpura, macrohematuria, nephrotic syndrome, mesangial GN; hemodialysis at 23; renal graft at 24; hematuria and proteinuria recurred at 26; mesangial GN; chronic allograft nephropathy; hemodialysis at 28; second renal graft at 36. A younger brother with complete C4 deficiency (details unavailable)	SS (C4B-C4B)	A30 B18 DR7	(104)
10	Intron 28, splice donor	g8127a (GT → AT) both C4B genes	6, F, Austrian/Italian^d^	SLE, hypertension, erythema of face, hands and arms; microhematuria, proteinuria, membranoproliferative GN; chronic renal failure; hemodialysis at 26; renal graft at 31	SS (C4B-C4B)	A30 B18 DR7	(104)
11	Intron 28, splice donor	As above	5, M, Austrian/Italian^d^	SLE, skin lesions, microhematuria, proteinuria, MPGN; hemodialysis at 16, cadaveric renal transplant at 18; chronic renal graft nephropathy at 23, hemodialysis at 24; meningitis – *Aspergillus fumigatus*	As above	As above	(104)
12	Intron 28, splice donor	As above	5, F, Austrian/Italian^d^	Hematuria and proteinuria, MPGN; facial maculopapular rash; biopsy-proven skin vasculitis; mental disorder, severe cerebral vasculitis	As above	As above	(104)
13	Exon 13	R559X	6, M, Moroccan^e^	SLE, malar rash, photosensitivity, discoid rash, ANA (1/1280), positive anti-Ro/SSA, proteinuria and microscopic hematuria, GN	L (C4A)	A2 B17 DRB1*07	(42)
14	Exon 13	As above	17, M, Moroccan^e^	Recurrent infections, hematuria	L (C4A)	A2 B17 DRB1*07	(42)
15	Exon 36	4-bp (GACT) insertion at codon 1555, Y1556X; both C4A and C4B	12, F, Algerian	Malar rash, ANA (1/1024); anti-Ro, anti-Sm; moderate renal disease, recurrent lung infections, bacterial meningitis; osteomyelitis; died at age 12 related to cardiopulmonary complications	LS (C4A-C4B)	A1 B17 DRB1*13	(42)

*^a–e^Individuals marked with matching superscripts are from the same family*.

*nk, age of onset is not known; GN, glomerulonephritis; MPGN, membranoproliferative glomerulonephritis; SLE, systemic lupus erythematosus*.

**Figure 5:**
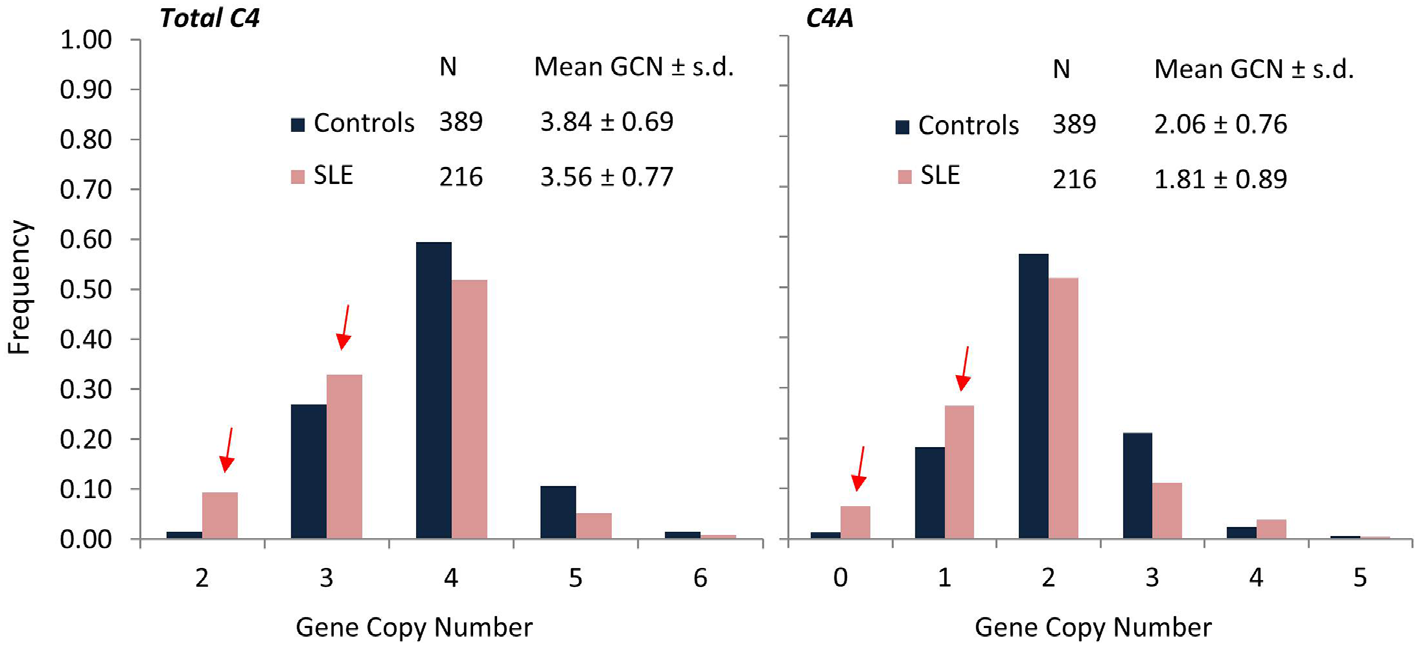
**Comparisons of frequencies for total C4, C4A, and C4B gene copy-number groups in SLE (red) and controls (blue)**. SLE patients (*N* = 216) of European ancestry showed significantly higher frequencies for lower copy-numbers of total C4 (GCN = 2 or 3) and C4A (GCN = 0 or 1) compared to healthy, race-matched controls (*N* = 389). Mean GCN for Total C4 in SLE (3.56 ± 0.77) was significantly lower than in controls (3.84 ± 0.69; *p* = 5.3 × 10^−6^, *t*-test). Similarly, mean GCN for C4A in SLE (1.81 ± 0.89) was significantly lower than in controls (2.06 ± 0.76; *p* = 2.0 × 10^−4^, *t*-test) [modified from Ref. (13)].

While a complete deficiency of C4 is rare, an isotype deficiency of either C4A or C4B is much more commonly observed and has been implicated in several autoimmune diseases (13, 110–113). To investigate the C4 genetic diversities in SLE, a study population consisting of 216 female SLE patients, 17 male SLE patients, 362 first degree relatives, and 389 unrelated healthy female controls, and 128 male controls was investigated rigorously (13). In the study group of European Americans, total gene copy-number (GCN) of C4 ranged from 2 to 6 copies, GCN of C4A ranged from 0 to 5 copies, and GCN of C4B ranged from 0 to 4 copies. In comparison to healthy controls, SLE patients had significant reductions of GCN of total C4 (Figure 5). Among the SLE patients, 9.3% had only two copies of C4 genes, compared to 1.5% in healthy controls. The effect size of SLE disease risk (or odds ratio) for subjects who had only two copies of C4 genes was 6.51. Of the two C4 isotypes C4A and C4B, there were no significant differences detected among GCN of C4B between SLE and controls. However, significant decreases of GCN of C4A were noted in SLE patients. Among SLE patients, 6.5% had a homozygous deficiency (i.e., 0 copy) of C4A and 26.4% had a heterozygous deficiency (i.e., 1 copy), compared to 1.3 and 18.2%, respectively, in healthy controls. The odds ratio for SLE for a subject with C4A homozygous deficiency of C4A was 5.27. In other words, a total C4 GCN = 2 or a C4A GCN = 0 (C4A deficiency) are large effect size genetic risk factors for human SLE. Moreover, family based association tests revealed that monomodular RCCX haplotypes with a single short C4B gene and C4A deficiency were more frequently transmitted to the SLE patients than normal heritance pattern (*p* = 0.005). To date, no other *common* genetic variant has been identified to be so strongly associated with SLE. Remarkably, 32.9% of SLE subjects carried the risk factor of low GCN of C4A. As with most common genetic variants associated with autoimmune disease, the risk factor is also present in the general population with considerable frequency (19.5% in this study).

From another way of statistical analysis, C4 GCN variations are continuous variations, and therefore, the mean of GCNs for total C4, C4A, C4B long genes and short genes can each be compared by Student’s *t*-test between patients and controls. The mean GCN (±SD) for total C4 between female controls and female patients were 3.81 ± 0.75 and 3.56 ± 0.77, respectively (*p* = 0.0001). The mean C4A GCN (±SD) was 2.05 ± 0.79 in controls and 1.81 ± 0.89 in SLE (*p* = 0.0005). The mean long C4 was 2.91 ± 1.03 in controls and 2.66 ± 1.14 in SLE (*p* = 0.005). In other words, when compared with controls, SLE showed reductions of 0.25 copies of total C4, 0.24 copies of C4A, or 0.25 copies of long C4. For C4B or short C4 genes, no significant differences were observed between the European American SLE and race-matched controls.

As the first original publication to document the highly prevalent, multi-allelic gene CNVs of complement C4 in SLE, this work (13) went through independent and rigorous data generation and validation processes to confirm the CNV calls. Those processes included (a) pulsed-field gel electrophoresis to resolve *Pme*I-digested genomic DNA fragments and Southern blot analysis for long-range mapping to elucidate the physical size of RCCX modules in haplotypes; (b) *Taq*I restriction digests and genomic Southern blot analyses (restriction fragment length polymorphisms) to resolve the relative dosages of RP1-C4L (7.0 kb), RP1-C4S (6.4 kb), RP2-C4L (6.0 kb), RP2-C4S (5.4 kb), plus relative dosages of CYP21B to CYP21A, and relative dosages of TNXB to TNXA; (c) *Psh*AI-*Pvu*II digests of genomic DNA and Southern blot analysis to segregate C4A and C4B and yield their relative dosages; (d) immunofixation of EDTA-plasma for polymorphic variants of C4A and C4B proteins resolved by high voltage agarose gel electrophoresis, based on differences in electric charges of C4 allotypes; and (e) corroboration of C4 genotypes and phenotypes of study subjects from data of family members.

Subsequently, TaqMan-based quantitative PCR amplicons for total C4, C4A, C4B long genes and short genes were developed and applied for replication studies, particularly when quantities of genomic DNA for patients and controls are limiting. In this later case, internal data validation is achieved when GCNs of total C4 = GCNs of C4A + C4B = GCNs of C4 long + C4 short. Such qPCR strategy is sensitive and highly robust when the quality of genomic DNA is excellent, which can be reflected by internal data validation of the independent amplicons. Our experience suggested that genomic DNA samples at low concentrations (≤15 ng/μl) are relatively unstable in storage, particularly if they had gone through rounds of freeze-thaws, and tend to yield inconsistent data in analyses of multi-allelic CNVs. This makes individual internal data validation crucial for data accuracy.

**Figure 6:**
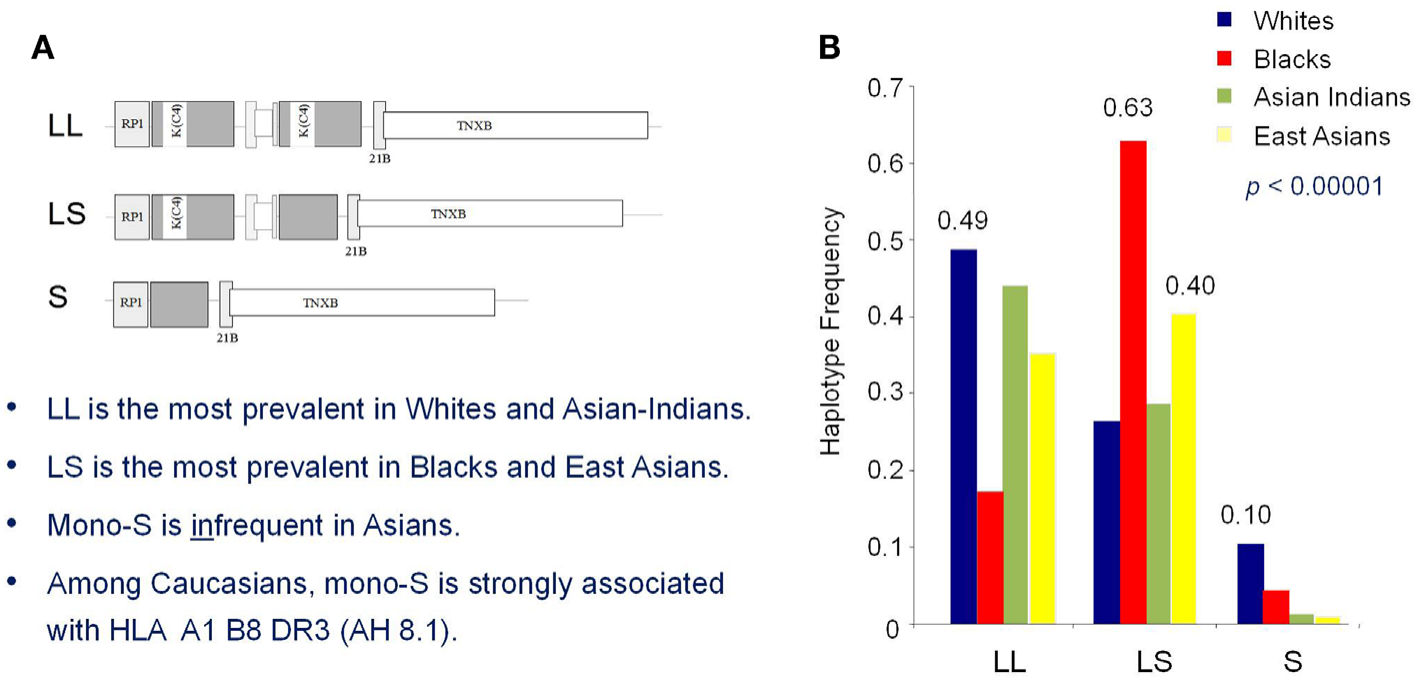
**Race-specific distribution patterns of RCCX modules in human populations**. The size dichotomy of C4 genes and copy-number variation of RCCX modules on an MHC haplotype together create a repertoire of length variants among different human subjects, which also exist with race-specific distribution patterns. **(A)** The most prevalent haplotypes of RCCX in Whites and Asian-Indians are the bimodular long-long (LL) and bimodular long-short (LS) in Blacks and East-Asians. **(B)** Notably, monomodular-short (mono-S or S) haplotypes with a single short C4B gene and C4A deficiency is relatively common in White and Black subjects but almost absent in Asians [modified from Ref. (118)].

Association of lower GCN of total C4 and C4A-deficiency as a risk factor of SLE have also been observed in three independent East-Asian studies (114–116). C4A deficiency in subjects of European ancestry is primarily attributed to the presence of a single short C4B gene (mono-S) in the HLA that is predominantly in linkage disequilibrium with HLA A*01, B*08, and DRB1*0301 that is dubbed ancestral haplotype 8.1 (AH8.1) (117). Intriguingly, such AH8.1 haplotype is basically absent among East-Asian subjects (Figure 6), supporting the evidence that C4A-deficiency association with SLE is *not* due to linkage disequilibrium with certain HLA haplotypes (e.g., DRB1*0301). Different mechanisms leading to C4A deficiency in East Asians include (a) monomodular RCCX with a single long C4B gene, (b) bimodular RCCX haplotypes encoding C4B1-C4B96 or C4B1-C4B1 are prevalent in Asian SLE (116). Certainly other genetic or environmental risk factors, combined with the C4A deficiency, contributed to SLE development in genetically predisposed patients. Still, it is of interest to examine if restoring C4A in such (C4A-deficient) patients would result in positive therapeutic outcomes.

On a recent case/control study of British SLE (cases, *N* = 501; controls, *N* = 719), total C4 GCNs were determined by a paralog ratio test, which employed a set of primers (16-mer) that hybridized to and PCR-amplified the duplicated regions of complement C4 in the MHC plus a unique and non-variable region in chromosome 19 as reference control (for two copies). C4A and C4B were deduced from the ratios of C4A and C4B that is determined by *Nla*IV restriction digest of a different PCR product spanning the C4-isotypic site (158 bp for C4B and 91 bp for C4A after *Nla*IV digest) and were resolved by capillary electrophoresis (119). While extensive GCN variations and associated polymorphisms were observable for complement C4, the difference on the mean copy number of total C4 between British SLE and controls was only marginal (3.79 ± 0.98 in SLE; 3.89 ± 0.98 in controls; *p* = 0.046). On the other hand, the mean copy numbers of C4A (1.82 ± 0.93 in SLE, 2.08 ± 0.93 in controls; *p* < 0.001) and C4B (1.96 ± 0.93 in SLE and 1.81 ± 0.72 in controls; *p* < 0.001) were both significantly reduced in SLE. However, in multiple logistic regression models, deficiencies of C4A or C4B both became insignificant in the presence of DRB1*03. This led the investigators to conclude that “…partial complement C4 deficiency states are not independent risk factors for SLE in UK….” It is notable that there were significant differences in the architecture of GCN group distributions between the British (119) and the US study populations (13).

Going through the previously mentioned Boteva study on C4-CNV determination, we note two issues that probably led to data misinterpretation. The first was a lack of an internal data validation for GCN calls. The second was an additional procedure to overcome the artifacts introduced by heteroduplex formation between multiple alleles during PCR, which would be resistant to restriction digest (*Nla*IV). Resistance to *Nla*IV digestions would therefore skew relative dosage of C4A and C4B and lead to misinterpretation of C4 copy-number calls (97, 120). As to dissecting the relative roles of C4-CNVs or C4A-deficiency and HLA-DRB1 alleles in SLE, the issues on the lack of internal validation for the reliability of C4-CNV calls cannot be ignored.

The unprecedented variations of GCNs with high frequencies of homozygous or heterozygous deficiency of C4A or C4B, and continuous variations in copy-numbers from one to four copies of C4 genes on each copy of chromosome 6 (or haplotype) among healthy subjects and SLE patients inevitably pose great challenges both technically and conceptually: the former for accurate data acquisition and the latter for accurate data interpretation. Without deliberate design and rigorous and independent validation strategies, unfortunately, many studies were inherently tainted with misinterpretations or partially correct or inappropriate conclusions. On determining the roles of common and multiallelic CNVs in health and disease such as those for complement C4A and C4B, immunoglobulin Fcγ receptors FCGR3A and FCBR3B, and neutrophil alpha-defensins DEFA3 and DEFA1, it is critically important to have meticulous experimental design and methods to acquire accurate and consistent data. Realization of the DNA sequence basis that causes a phenotype or functional diversity is essential for specific experimental method. As mentioned above, the heteroduplexes issue during the PCR process needs to be considered and resolved. Moreover, genetic studies of a complex disease usually require hundreds to thousands of genomic DNA samples from cases and controls, which are generally obtained from multiple sources. For determination of common and continuous CNVs, the high quality of genomic DNA samples is essential. Heterogeneous quality of DNA samples has a high tendency to yield inconsistent data. Under those conditions, independent replication and rigorous internal validation methods of samples from every subject becomes a necessity.

### Complement C2 Deficiency

The complement C2 gene is located in the HLA class III region on the short arm of chromosome 6. Serum C2 is a precursor protein that is cleaved by activated C1 into two fragments: C2b and C2a. C2a is a serine protease and forms the C3 convertase along with C4b (denoted C4b2a) (38, 121). C2 also functions as a critical component in the lectin pathway. MBL or ficolins in complex with MASP-1 bind to relevant carbohydrate molecules and activate MASP-2, which then cleaves C2 and C4, forming a C3 convertase identical to that formed in the CP (122). Overall, C2 functions as a key component in the classical and lectin pathway, thereby providing defense against microbial infection and assisting in removal of immune complexes.

Among individuals of European descent, C2 deficiency occurs with an estimated prevalence of 1/20,000, which probably accounts for <1% of SLE patients. There are two types of C2 deficiency (123, 124). Type 1 C2 deficiency is caused by non-sense mutations leading to the absence of protein biosynthesis. The predominant form of such type 1 deficiency was a 28-bp deletion that removed 9 bp from the 3′ end of exon 6 and 19 bp from the 5′ end of intron 6 in the C2 gene, leading to a skipping of exon 6 in the C2 mRNA and generation of premature stop codon (123). Such 28-bp deletion is present in the HLA haplotype with A10 (A25) and B18 in the class I region, BF-S, C2Q0, C4A4, and C4B2 in the class III region, and DRB1*15 (DR2) in the class II region. The second form of Type 1 deficiency is present in HLA A3, B35, DR4, BF-F, C2Q0, C4A3, and C4A2 (125). The cause is a 2-bp deletion in exon 2 of C2 gene that leads to a non-sense mutation.

About 10% of C2 deficiency is secondary to the Type II deficiency in which the C2 protein is synthesized but not secreted. The molecular defects identified as missense mutations are C111Y, S189F, and G444R (126, 127). It is not clear how these mutations block the secretion of C2 protein.

Unlike a deficiency of proteins for the C1 complex or C4 described earlier, the penetrance of C2 deficiency on SLE is about 10%. Similar to other risk factors for SLE, there is a female predominance for patients with C2 deficiency. C2-deficient SLE patients tend to have early childhood onset but a milder disease process with prominent photosensitive dermatologic manifestations, speckled ANAs (the autoantibody specificity is common for the Ro/SSA antigen), and a family history of SLE. Anti-DNA antibody tests are usually negative, and severe kidney disease is rare.

## Acquired Deficiencies and Autoantibodies to Complement Components

Autoantibodies have been reported that bind with high affinity to complement proteins, particularly in SLE patients (128). Most of these antibodies are not directed against native proteins, but instead directed against neoepitopes. Such epitopes becomes exposed in active or inactivated proteins or upon multi-molecular complex assembly in the activation process or following proteolytic cleavage. The binding of these autoantibodies to complement proteins could lead to a state of an acquired deficiency and contribute to disease pathogenesis similar to the way genetic deficiencies do so. We will describe a series of autoantibodies that have been detected in systemic autoimmune disease against early components and regulators of the CP.

### Anti-C1 Autoantibodies

Approximately 30% of SLE patients synthesize autoantibodies against C1q. Their presence correlates with anti-dsDNA, nephritis, and low C4 and C3 in about 75% of such patients (129–131). A relevant question is whether the anti-C1q amplify the complement activation by immune complexes. The development of anti-C1q antibodies may arise in response to activation of the CP. Following CP activation, C1q remains attached to immune complexes and therefore is located at the site of inflammation. Proteases at the inflammatory site may degrade IgG and C1q (autoantigen), generating multiple proteolytic fragments of IgG and C1q. This may be an explanation for C1q antibodies and anti-IgG and anti-IgM (rheumatoid factors) that develop in SLE patients.

A large study was recently reported in which one objective was to assess the specificity of anti-C1q antibodies and their associations with SLE manifestations and diagnostic tests (132). The authors confirmed the association of anti-C1q antibodies, low complement (C4 and C3), and anti-dsDNA antibodies. Further, this combination had the highest serological association with renal disease. Anti-C1q antibodies were detected in 28% of all SLE patients, but were observed more frequently in patients with renal disease in ~68%. Anti-C1q autoantibodies were observed in 5–10% of patients with related systemic rheumatic diseases. It was suggested that the presence of anti-C1q antibodies contributes to a nephritis flare (130).

The presence of autoantibodies against other components of the C1 complex is less well established. A report of 15 SLE patients demonstrated that seven of them had autoantibodies to C1s (133). The binding of these antibodies to C1s was shown to *enhance* its enzymatic activity for C4, providing possible additional explanation for low serum C4.

### Anti-C1 Inhibitor Autoantibodies

An IgG autoantibody that inactivates C1-inhibitor (anti-C1-Inh) was initially described in a patient with the acquired angioedema (AAE) syndrome that mimics hereditary angioedema (134). Anti-C1-Inh autoantibodies have also been described in SLE, especially those exhibiting symptoms of angioedema (135, 136). Anti-C1-Inh antibodies bind and inactivate C1-Inh so that it is no longer available to participate in the regulation of C1. As a result, the CP is excessively activated, leading to development of angioedema as well as possibly more severe renal diseases. A more recent study of 202 SLE patients and 134 healthy controls detected anti-C1-Inh autoantibodies in 17% of SLE patients and 4% of controls (137). In SLE patients, the anti-C1-Inh levels correlated with the duration and activity of SLE but did not correlate with SLE laboratory parameters, including serum levels of C3 and C4. Conversely, 1–2% of hereditary angioedema patients develop SLE, probably related to the chronically very low C4 and C2.

### C3 and C4 Nephritic Factors

C3 and C4 nephritic factors are IgG autoantibodies that bind to and stabilize the AP C3 convertase and the CP C3 convertase, respectively. By binding the C3 convertases, C3 and C4 nephritic factors prolong the half-life by preventing the regulation of C3 convertases. This results in uncontrolled complement activation and increased consumption and depletion of serum C3. C3 and C4 nephritic factors are associated with membranoproliferative glomerulonephritis, acquired partial lipodystrophy, and post-infectious acute glomerulonephritis (138–140). Both of these autoantibodies have been detected in SLE patients, and suggested to be associated with renal disease, but their prevalence and role in pathogenesis of systemic autoimmune disease is not well documented (139–141).

## Therapeutic Potentials of Complement Abnormalities in Autoimmune Disease

Because the complement system is increasingly found to be associated with autoimmune diseases, it is an attractive therapeutic target. However, as discussed earlier, complement deficiencies are overwhelmingly associated with increased susceptibility to autoimmune disease, most notably SLE. Therefore, at first glance, the idea that treatment with complement inhibitors would provide a *general* relief in such diseases seems illogical. Processing of immune complexes is facilitated by deposition of complement fragments, the ensuing binding of complement-decorated immune complexes to CR1 on erythrocytes, and transfer to resident monocytes or macrophages for destruction and/or antigen presentation. For that reason, blocking any step up to and including C3 could inhibit immune complex processing and possibly exacerbate disease in autoimmune patients. Moreover, inhibiting the complement system would further enhance susceptibility to infections, on top of the increased frequency of infections already observed in patients under immunosuppressive therapy.

On the other hand, complement activation is quite evident and partly responsible for tissue damage seen in autoimmune patients with established disease. In lupus, key mediators of tissue damage include C4b/C3b, C5a/C3a, and the MAC, all of which modulate membrane integrity or trigger inflammation in a setting where autoantibodies are already present in large amounts. Therapeutic goals should be aimed at inhibiting this cellular injury and preventing production of the proinflammatory peptide fragments. In atypical hemolytic uremic syndrome (aHUS), endothelial damage is mediated by over-activation of the AP. Inhibition of C5 cleavage has proven to be an effective therapy (142). Anti-C5 therapy has been effective in treatment of antigen-induced arthritis or experimental autoimmune uveoretinitis in animal models (143, 144). Other studies in animal models of arthritis that showed success in reducing inflammation and preventing disease progression include treatment with CR1 (145), antagonists of C3a receptor (146), and the complement regulator CD59 (147). Soluble CR1 prevented dysregulation of C3 convertase in sera from dense deposit disease patients (148). The same study reported that short-term use of soluble CR1 in a pediatric patient with end-stage renal failure normalized the activity of the terminal complement pathway. Inhibition of complement activation specifically in SLE with renal diseases deserves to be assessed.

Another treatment approach is to replenish complement proteins in patients with a complete complement deficiency. As discussed earlier, complete genetic deficiencies of complement are quite rare, but isotype deficiency and functional or acquired deficiencies are quite common. An obvious approach seems to be supplement or replacement of the missing component, systematically or locally. Currently, however, purified or recombinant complement proteins are not available for treatment purposes. One report indicated successful treatment of a C2-deficient patient with SLE using whole plasma preparations (149). Moreover, in patients with established disease and low C4 or C3, an increase in C4 and C3 is associated with a favorable response to treatment.

Fresh frozen plasma is able to restore C1q activity in C1q-deficient patients temporarily, but such activity drops off rapidly within 2 weeks. Thus, weekly infusions of plasma become necessary, which is burdensome, and confers its own risk of infections and thrombotic complications (69). Unlike most other component proteins of complement in blood, the primary site of biosynthesis for C1q is *not* in the liver, but rather in myeloid cells including macrophages, monocytes, and dendritic cells, which originate in the bone marrow. The effect of restoring the C1q protein to reconstitute complement function has been tested in mouse models. Bone marrow transplantation (BMT) of hematopoietic stem cells from wild-type animals has been shown to be effective in treating C1q deficient animals (150, 151).

Recently, BMT for hematopoietic stem cell therapy (HSCT) has been performed in a single case of a Pakistani C1q-deficient patient (152). In this particular case, involving a consanguineous family, the father and five of his six sons had C1q-deficiency. The father died of chronic glomerulonephritis at age 38, and one of his sons died at age 17 months from *Streptococcus pneumonia* meningitis. Another son was incapacitated by a CNS vasculopathy at 17 years of age. The index patient is the third son who survived *S. pneumonia*-induced meningitis at age 3 but developed an acute CNS vasculopathy at age 10. At age 16, his disease was brought under control through treatment with intravenous cyclophosphamide (pulse therapy) and B-cell depletion with rituximab. However, he had a persistent lupus rash and increased levels of anti-Ro (SSA-60) in addition to anticardiolipin antibodies. In view of his poor prognosis, HSCT was performed using bone marrow from his HLA-matched healthy brother, with graft-versus-host disease prophylaxis. Restoration of hematopoiesis, myelopoiesis, platelet production, and complement function with normal levels of C1q and CH50 were observed 2–4 weeks after transplantation. Such a result gives hope to SLE-patients with a C1q deficiency and a severe clinical course, although further studies are desirable.

## Early Complement Components in Other Autoimmune Diseases

Complement activation products and genetic polymorphisms have been observed and shown to contribute to inflammation and tissue damage of autoimmune diseases besides SLE. We will briefly summarize findings of complement activation and involvement in a few other autoimmune diseases.

### Antiphospholipid Syndrome

Anti-phospholipid syndrome (APS) is characterized by arterial or venous thrombosis and recurrent pregnancy loss. The complement profile in general parallels that observed in SLE with CP activation being predominantly involved (153–156). Hypocomplementemia has been associated with fetal loss, pre-term delivery, and low-birth weight (157). Serum complement levels were significantly lower in patients with primary APS compared to patients with APS associated with a connective tissue disease (155). The patients with primary APS also had higher levels of complement activation fragments C3a and C4a demonstrating that the hypocomplementemia is, as expected, due to complement consumption (154, 155).

### Dermatomyositis

Of the inflammatory myopathies, dermatomyositis (DM) is the one most reported to possibly be associated with a complement-mediated pathogenesis (158). Complement-mediated destruction of perivascular endothelium and perifascicular ischemia of muscle fibers in biopsies from DM patients have been demonstrated by multiple investigators (159–164). Circulating immune complexes, IgG and IgM, complement C3, and late components of complement activation C5b–C9 MAC were detected in DM muscle and skin biopsies. A single case of an individual with a complete C2 genetic deficiency was reported to have DM (165). The observation of complement components and complement fragments indicates the possible involvement of complement pathways in tissue damage. Previous studies revealed that HLA class II gene DRB1 allele *0301 (also known as DR3) is the major immunogenetic risk factor for juvenile DM (JDM) (166–170). However, also present in the HLA region is the locus for complement C4. Specifically, DR3 in European subjects is in strong linkage disequilibrium with a particular C4 haplotype: a single C4B gene but the absence of a C4A gene (118, 171). A recent study found that C4A deficiency was independent of DR3 in association with JDM in a study of 95 patients and 500 race-matched controls of European ancestry (113). In addition, the authors demonstrated through multiple logistic regression analyses that the concurrence of C4A deficiency and DR3 together contributed toward the highest risk of JDM with an odds ratio of 3.2.

### Rheumatoid Arthritis

Several autoantibodies that play a major role in the autoimmune attack of synovia have been identified in rheumatoid arthritis (RA). There are three major types of RA-associated autoantibodies (i) anti-cyclic citrullinated peptide/protein autoantibodies (anti-CCP), (ii) autoantibodies against the Fc-fragments of IgG (rheumatoid factor, RF), and (iii) anti-type II collagen or glucose-6-phosphoisomerase autoantibodies (anti-G6PI). All of these autoantibodies bind their respective self-antigen, leading to increased levels of ICs and activation of the CP observed in RA patients (172–174). CP activation in RA patients has also been reported to be mediated by C-reactive protein (CRP) or fibromodulin (175, 176). Complexes formed between CRP and activated complement components were increased in the majority of RA patients and were further increased in patients with active disease versus patients with inactive disease. Agents/components that mediate and trigger inflammation in RA are likely derived from cartilage since disease activity subsides after joint replacement. Sjoberg et al. showed that the cartilage component fibromodulin can activate complement specifically by binding C1q, thereby activating the C1 complex and the remainder of the complement cascade (176). The evidence of complement activation in synovial fluid and the identification of several complement-triggering agents in RA support complement-mediated disease pathogenesis.

Depressed levels of complement proteins and elevated levels of complement cleavage products and late components MAC C5b–C9 were noted in tissue specific to RA pathogenesis, such as synovial fluid (177–180). Furthermore, the subsequent generation of anaphylatoxins C3a and C5a in synovial fluid creates an inflammatory state that attracts and activates neutrophils and other myeloid cells to the site of complement activation in the inflamed joints. The harmful effects of complement activation in RA are remarkable and evident. A recent study of C4 genetics in RA patients revealed that a deficiency of C4B genes was significantly more frequent in RA patients compared to non-RA patients or healthy controls (110). In other words, 40% of RA patients had a C4B deficiency compared to only 21.6% of controls, resulting in an odds ratio of 2.99 for RA subjects. Replication cohorts of RA patients are needed to validate and strengthen the possible association of C4B deficiency with RA, which could be secondary to the prevalence of HLA-DR4.

### Sjögren’s Syndrome

As with many autoimmune diseases, there are a number of autoantibodies that have been reported in primary Sjogren’s syndrome (SS). Therefore, complement activation via the CP may be involved in the development and/or pathogenesis of primary SS. A frequent clinical observation in primary SS is hypocomplementemia, particularly low serum levels of C3 and C4. Some studies reported that in the most severe cases of primary SS, the *complement inhibitor C4b-binding protein was decreased* in parallel with C3 and C4 levels, suggesting that dysregulation of complement may mediate disease pathogenesis (181). It is generally hypothesized that low C3 and C4 levels are a result of consumption by CP activation mediated by ICs in primary SS patients. Recent studies have showed that lymphoproliferative disease, mortality rates, and other severe disease manifestations were significantly higher in patients with low levels of C3 or C4 (182–184). Therefore, complement levels may be more a marker or predictor of disease activity in established SS patients.

## Conclusion

A complete genetic deficiency in any one of the early components engaged in the CP of complement activation, C1q, C1r, C1s, or C4 almost always leads to SLE in humans, irrespective of race or sex. This phenomenon underscores the necessity for all of these early complement components, probably acting in concert, to achieve immune tolerance or prevent autoimmunity. While the incidence of a homozygous deficiency for one of these early acting complement components is extremely rare, partial genetic deficiency due to gene CNV of complement C4A (and total C4) and acquired deficiency such as secondary to anti-C1q autoantibodies are common. In combination, inherited insufficiency and acquired deficiency for early components of the CP may exist in over half of SLE patients with European ancestry. On the other hand, high copy numbers of total C4 and C4A are prevalent among healthy subjects and protective against SLE. Although less conclusively demonstrated, less explored topics include the single nucleotide polymorphisms (SNPs) in those complement genes that may modulate functional protein activities or gene expression levels, the presence of other complement autoantibodies or nephritic factors, dysfunctional regulatory proteins, or toxic side effects of drugs that may also contribute to inappropriate complement activation.

Genetic insufficiency and acquired deficiency of complement have medium to large effect size on disease susceptibility in European SLE. The frequency and effect size of specific genetic variants for a complex disease tend to be race specific. Considering the high prevalence of renal disease in African, Asian, and Hispanic SLE patients, it is of interest to further extend investigations on the roles of complement risk factors in these racial/ethnic groups.

Fluctuations in either hemolytic complement activity or levels of serum C3 and C4 in SLE patients were noticed over half of a century ago. More recently, high levels of processed C4d activation product attached to multiple cell types of hematopoietic origin are present in most SLE patients. Comprehensive data on genetic and acquired risk factors, plus cross-sectional and longitudinal profiling of autoantibodies and serum levels of native and activated complement proteins, and cell-bound levels of processed activation products hold promise as more sensitive biomarkers for SLE. Additionally, similar investigations should be extended to other autoimmune or inflammatory diseases including Sjögren’s syndrome, DM, RA, and antiphospholipid syndrome.

## Author Contributions

KL, YW, YY, CS, GH, LH, JA, and CYY all contributed to the acquisition of data, drafting and revision of the content, and final approval of the version to be published.

## Conflict of Interest Statement

The authors declare that the research was conducted in the absence of any commercial or financial relationships that could be construed as a potential conflict of interest.
